# The relationship of personality, alexithymia, anxiety symptoms, and odor awareness: a mediation analysis

**DOI:** 10.1186/s12888-024-05653-y

**Published:** 2024-03-06

**Authors:** Binfeng Zhang, Xiuxia Li, Hongzhen Deng, Peixuan Tan, Wanyong He, Shuling Huang, Lu Wang, Hao Xu, Lei Cao, Guanghui Nie

**Affiliations:** 1 Department of Psychology, Guangxi Medical University, Nanning, China; 2 School of Health Management, Southern Medical University, Guangzhou, China; 3 School of Public Health, Guangxi Medical University, Shuangyong Road 22, 530021 Nanning, Guangxi China

**Keywords:** Personality, Alexithymia, Anxiety, Odor awareness, Limbic system, Mediation

## Abstract

**Objective:**

Personality, emotions, and olfaction exhibit partial anatomical overlap in the limbic system structure, establishing potential mechanisms between personality, affective disorders, and olfactory-related aspects. Thus, this study aims to investigate the associations among the Big Five personality traits, alexithymia, anxiety symptoms, and odor awareness.

**Methods:**

A total of 863 college participants were recruited for this study. All participants completed the Chinese Big Five Personality Inventory-15, the Odor Awareness Scale (OAS), the Toronto Alexithymia Scale-20, and the Generalized Anxiety Disorder Screener-7. Structural equation modeling was employed to examine the hypothesized mediated model.

**Results:**

The findings revealed the majority of significant intercorrelations among the dimensions of the Big Five personality traits, alexithymia, anxiety symptoms, and OAS (|*r*| = 0.072–0.567, *p* < 0.05). Alexithymia and anxiety symptoms exhibited a serial mediation effect between neuroticism and OAS (95%CI[0.001, 0.014]), conscientiousness and OAS (95%CI[-0.008, -0.001]), and extraversion and OAS (95%CI[-0.006, -0.001]). Anxiety symptoms mediated the relationship between agreeableness and OAS (95%CI[-0.023, -0.001]) and between openness and OAS (95%CI [0.004, 0.024]).

**Conclusion:**

The mediating roles of alexithymia and anxiety symptoms between the Big Five personality traits and odor awareness support the idea of a certain level of association among personality, emotions, and olfaction, with the underlying role of the limbic system structure. This enhances our understanding of personality, emotions, and olfaction and provides insights for future intervention measures for affective disorders and olfactory dysfunctions.

## Introduction

The limbic system maintains close associations with emotions and olfaction. The limbic system encompasses cerebral structures that have evolved from the paleocortex and the archicortex, along with closely related neural structures and cerebral nuclei. It includes the limbic lobe and subcortical structures intricately linked to it, such as the amygdala, septal nuclei, insular cortex, cingulate gyrus, hypothalamus, anterior thalamic nuclei, and the roof of the midbrain [1]. The physiological characteristics of the limbic system establish a profound connection between olfaction and emotions, as there exists an anatomical overlap between olfactory and emotion-related regions within the limbic system [2–4]. Central structures involved in the processing of olfactory signals primarily comprise the amygdala, hippocampus, orbitofrontal cortex, insula, and cingulate gyrus. These regions concurrently process emotional signals from other sensory modalities, thereby laying the foundation for the interplay between olfaction and emotions [5]. It has been confirmed that olfactory impairments are particularly associated with neuropsychiatric disorders, especially those characterized by significant affective symptoms, such as schizophrenia, bipolar disorder, depression disorder, anxiety disorder, etc. [6–11].

Odor awareness refers to an individual’s proclivity to rely on olfactory stimuli in guiding attitudes and behaviors [12], reflecting the extent of their attention to odors [13–15]. Research indicates that individuals with low odor awareness exhibit significantly diminished olfactory performance, including olfactory threshold, recognition, and discrimination, compared to those with high odor awareness [12, 16]. Those with heightened odor awareness demonstrate superior olfactory recognition memory and are capable of identifying a wider array of odors than their counterparts with low odor awareness [17]. Consequently, odor awareness can serve as an indirect index of one’s olfactory performance, reflecting a certain degree of olfactory performance [12, 15, 16]. Given olfactory performance relates to odor awareness and affective disorders, studies have delved into the mechanistic relationship between affective disorders and odor awareness. It has been discovered that individuals afflicted with panic disorder exhibit higher levels of odor awareness compared to healthy individuals in control groups [18, 19]. Among non-clinical populations, individuals displaying symptoms of anxiety and neurotic personality traits exhibit heightened olfactory sensitivity and reactivity compared to healthy individuals [20–25]. In a recent study [15], anxiety symptoms were found to positively predict odor awareness, while alexithymia negatively influenced odor awareness; additionally, the interaction between alexithymia and social anxiety symptoms had a significant impact on odor awareness; furthermore, social anxiety symptoms and alexithymia emerged as negative predictors of social odor awareness on body odors specifically [26], while depressive symptoms emerged as a positive predictor of social odor awareness. Consequently, affective disorders or symptoms may have a profound and intricate association with odor awareness.

The limbic system is not only related to olfaction and emotion, but also closely related to personality. Personality neuroscience research suggests that there may be neurobiological mechanisms behind the Big Five personalities, related to the limbic system [27]. Studies have indicated that extraversion predicts neural activity in the amygdala [28–30] and the nucleus accumbens [31], and there is a positive correlation between the amygdala and other brain regions with extraversion [32]. Neuroticism stands as a primary personality risk factor within psychopathology [33]. Research demonstrates that neuroticism predicts activation in the insula [31] and is associated with amygdala activity [34–36]. Additionally, neuroticism positively correlates with the volume of the amygdala [37–40] and shows significant associations with the hippocampus [41]. A structural magnetic resonance study has revealed a negative correlation between conscientiousness and white matter volume in regions such as the insula, the caudate nucleus, the cingulate cortex, and the prefrontal cortex [42]. Furthermore, some studies have found associations between conscientiousness and the anterior insula and the anterior cingulate cortex [42–47]. Agreeableness may be linked to emotional regulation, as research has indicated that agreeableness predicts the inhibition of aggressive impulses and other socially disruptive emotional behaviors [48]. In a longitudinal study, amygdala volume at the age of 26 was negatively correlated with current levels of aggression and a history of aggression [49]. An MRI study found a relationship between agreeableness and the volume of brain regions associated with social information processing, such as the posterior cingulate cortex [50]. As for openness, it has been reported that a positron emission tomography (PET) study has found that openness correlates with neural activity of the anterior cingulate cortex in the resting state [51]. Therefore, it is reasonable to speculate that the Big Five personality traits may be associated with olfactory tasks or activities and affective disorders or symptoms, given the underlying limbic system.

Existing research has illuminated that personality traits (neuroticism, impulsivity, and self-confidence deficiency) stand as potent predictors of olfactory recognition, even when controlling for individual differences such as age, gender, education level, and overall cognitive function [52]. Olfactory-impaired individuals exhibit elevated levels of neurotic traits compared to those with normal olfactory functions [53]. Furthermore, a study has indicated a modest yet significant positive correlation between agreeableness and olfactory sensitivity [54]. Additionally, research has revealed associations between personality traits and olfactory attitudes, with individuals constrained by societal demands (e.g., hypocrisy) relying more on olfaction for daily decision-making [55]. These findings suggest a potential link between personality traits and odor awareness. Regarding the intersection of personality and affective disorders, studies have substantiated a clear relationship between the Big Five personality dimensions and affective disorders [56]. A meta-analysis has demonstrated associations between the Big Five personality dimensions and anxiety and depressive disorders [57]. Notably, neuroticism has been closely linked to symptoms of anxiety and depression. Furthermore, research indicates that alexithymia plays a mediating role in the relationship between the big five personality traits and mental health [58]. Hence, the Big Five personality may also have some associations to affective disorders and odor awareness.

This is an exploratory study. In keeping with the theory above, there may be some mechanism between personality traits, affective symptoms, and odor awareness. According to the existing research results, affective symptoms may be in an intermediate position between personality traits and odor awareness. However, upon conducting a comprehensive review of the literature, we have identified a limited number of studies addressing the relationship between affective disorders or symptoms and odor awareness, with only a few such investigations currently available [15, 18, 19]. Concerning research on the connection between personality and odor awareness, the academic community has yet to furnish any relevant reports. Hence, we embark on an exploration of the associations between the Big Five personality traits, alexithymia, anxiety symptoms, and odor awareness, seeking to uncover the role played by the limbic system in these dynamics. We formulated the following hypotheses:

### Hypothesis 1

The Big Five personality traits, alexithymia, anxiety symptoms, and olfactory awareness would mutually intercorrelate.

### Hypothesis 2

Alexithymia and anxiety symptoms would mediate the relationship between the Big Five personality traits and odor awareness.

## Methods

### Participants

Participants were recruited from Guangxi Medical University in China and distributed anonymous paper questionnaires to students during their break time. After explaining the purpose of the survey, students voluntarily participated in the research. Students who completed the survey received 5 points for their regular grade. The eligibility criteria for participants were ages ranging from 18 to 45 years to minimize age-related olfactory variability. Exclusion criteria were as follows: (1) acute respiratory infections; (2) pregnancy or breastfeeding;(3) chronic obstructive pulmonary disease or nasal surgery; (3) neurological disorders; (4) unstable mental illnesses or other conditions that may affect olfaction function. We distributed 900 questionnaires and received 890 responses. After removing 27 questionnaires with missing data or failing lie detection questions, we were left with 863 valid responses. The sample consisted of 230 males (26.7%) and 633 females (73.3%) with an age range of 18 to 37 years (*M* = 22.311, *SD* = 2.055). All participants signed informed consent. The implementation of this study complied with the principles of the Helsinki Declaration and was approved by the Ethics Committee of Guangxi Medical University.

### Measures

#### The Chinese big five personality inventory-15(CBF-PI-15)

CBF-PI-15 is devised for the assessment of the five dimensions of the Big Five personality model [59]. CBF-PI-15 comprises 15 items, distributed across five dimensions, with three items per dimension. Responses to each item are rated on a Likert scale of 6 points, ranging from 1 (“strongly disagree”) to 6 (“strongly agree”). In this study, the Cronbach’s α coefficients for each dimension were as follows: 0.886 (neuroticism), 0.741 (conscientiousness), 0.887 (agreeableness), 0.862 (openness), and 0.771 (extraversion).

#### The odor awareness scale (OAS)

The OAS serves as an instrument to assess variations in an individual’s awareness of odor in their surrounding environment, which reflects the characteristics of how an individual processes olfactory information, responds to these stimuli, and the degree of attention paid to odors [12–15]. In this study, we employed the revised Chinese version of the OAS by Zhang et al. [60], comprising 27 items organized into three factors: “odor sensitivity,” “odor impact,” and “odor attention.” Each item is rated on a 5-point Likert scale, with higher total scores indicating heightened odor awareness. In this study, the Cronbach’s α coefficient for the OAS was 0.909.

#### The toronto alexithymia scale (TAS-20)

The TAS-20, developed by Bagby et al. [61], is employed to gauge the severity of alexithymia. This scale comprises 20 items delineated into three dimensions: difficulty in identifying feelings and distinguishing them from bodily sensations of emotions, difficulty in describing feelings to others, and an externally oriented cognitive style of thinking. The scale utilizes a 5-point Likert scale (1 = strongly disagree, 5 = strongly agree) for scoring, with five items reverse-scored (namely, items 4, 5, 10, 18, and 19). A higher total score indicates a greater degree of alexithymia. In this study, we utilized the Chinese version of the TAS-20 translated and revised by Zhu et al. [62]. The Cronbach’s α coefficient for the TAS-20 was 0.846.

#### The generalized anxiety disorder screener-7 (GAD-7)

The GAD-7, pioneered by Spitzer et al. [63], serves as a screening instrument employed to appraise the magnitude of generalized anxiety symptoms. Comprising seven items, it is designed to assess how often patients have been bothered in the past two weeks including feeling nervous and worried. Response choices span from “not at all” to “nearly every day,” and are set with scores of 0, 1, 2, or 3. In this research, we utilized the Chinese version of the GAD-7, as translated and refined by Xiaoyan et al. [64]. The Cronbach’s α coefficient for the GAD-7 was 0.932.

### Statistical analysis

Descriptive analysis of the data was performed using SPSS 27 software, and the interrelationships between variables were examined through the Pearson product-moment correlation coefficient. To construct a structural equation model, Mplus 8.3 software was employed, and the model fit was assessed using multiple fit indices [65–68]: Normed Chi-square (χ^2^/df), Comparative Fit Index (CFI), Tucker-Lewis Index (TLI), Standardized Root Mean Squared Residual(SRMS), and Root Mean Square Error of Approximation (RMSEA). Non-parametric BcBootstrap resampling was conducted 1000 times for the mediation analysis, and a 95% confidence interval excluding 0 indicated significant mediation effects. The mediation effect size was estimated by the absolute value of the ratio between the indirect and direct effects [69]. The statistical significance level was set at *p* < 0.05.

## Results

### Correlation analysis

The descriptive statistics for each variable and the correlation analysis are presented in Table 1. The correlation analysis revealed a mild negative association between OAS scores and Alexithymia scores (*r* = -0.088, *p* < 0.01), while OAS scores exhibited a positive correlation with Anxiety scores (*r* = 0.187, *p* < 0.001). Furthermore, OAS scores were significantly correlated with most of the Big Five personality dimensions: neuroticism (*r* = 0.180, *p* < 0.01), conscientiousness (*r* = 0.136, *p* < 0.01), agreeableness (*r* = 0.072, *p* < 0.05), openness (*r* = 0.152, *p* < 0.01), except for extraversion (*r* = -0.015, *p* > 0.05). Alexithymia scores demonstrated a positive correlation with Anxiety scores (*r* = 0.373, *p* < 0.01). Moreover, Alexithymia scores exhibited significant correlations with all Big Five personality dimensions: neuroticism (*r* = 0.480, *p* < 0.01), conscientiousness (*r* = -0.191, *p* < 0.01), agreeableness (*r* = -0.155, *p* < 0.01), openness (*r* = -0.148, *p* < 0.01), and extraversion (*r* = -0.255, *p* < 0.01). Anxiety scores were significantly correlated with most Big Five personality dimensions: neuroticism (*r* = 0.567, *p* < 0.01), conscientiousness (*r* = -0.074, *p* < 0.05), agreeableness (*r* = -0.170, *p* < 0.05), extraversion (*r* = -0.174, *p* < 0.01), except for openness (*r* = -0.027, *p* > 0.05). There were also mild intercorrelations among the Big Five personality dimensions. In addition, we analyzed the possible effects of gender and age on the three dependent variables in the mediation model. Employing a t-test for gender-based grouping, the results indicated that gender had no significant impact on alexithymia (*t* = 1.499, *p* > 0.05) and anxiety (*t* = -0.884, *p* > 0.05). Gender exhibited a significant effect on OAS (*t* = -2.621, *p* < 0.05), with a Cohen’s d of 0.202 which is considerably smaller than the minimum effect size of 0.41 for social science data [70]. Regarding age, the results showed no significant correlation between age and OAS (*r* = -0.034, *p* > 0.05) or anxiety (*r* = -0.064, *p* > 0.05), but the correlation between age and alexithymia was significant (*r* = -0.195, *p* < 0.05), while the effect size of which remained small [70, 71]. Consequently, gender and age were not included in subsequent mediation analysis.

**Table 1:** Descriptive statistics and correlation analysis

Variables	M	SD	1	2	3	4	5	6	7
1. OAS	93.433	14.180	-						
2. Alexithymia	52.893	9.321	-0.088^**^	-					
3. Anxiety	5.110	4.663	0.187^**^	0.373^**^	-				
4. N	8.793	3.701	0.180^**^	0.480^**^	0.567^**^	-			
5. C	11.531	2.836	0.136^**^	-0.191^**^	-0.074^*^	-0.007	-		
6. A	12.969	2.827	0.072^*^	-0.155^**^	-0.170^**^	-0.136^**^	0.255^**^	-	
7. O	9.596	3.214	0.152^**^	-0.148^**^	-0.027	-0.121^**^	0.225^**^	0.205^**^	-
8. E	9.875	3.124	-0.015	-0.255^**^	-0.174^**^	-0.226^**^	0.065	0.130^**^	0.236^**^

*Note* OAS = Odor Awareness Scale; N = neuroticism; C = conscientiousness; A = agreeableness; O = openness; E = extraversion

^*^p < 0.05, ^**^p < 0.01

### Structure equation modeling

To optimize the model, during the construction of the structural equation model, we employed the parceling method to aggregate indicators of latent variables, which is a recommended approach for model construction [72–74]. Both OAS and TAS-20 consist of three subscales, each of which was parcelled into a single indicator. GAD-7, without subscales, was parcelled into three indicators using the factorial algorithm [72, 75]. Each dimension of the Big Five personality traits was assessed with three indicators. The model fit indices included χ2/df = 3.815, CFI = 0.944, TLI = 0.931, SRMR = 0.057, RMSEA = 0.057 (90%CI[0.053, 0.061]), indicating a well-fitting model.

**Fig. 1:**
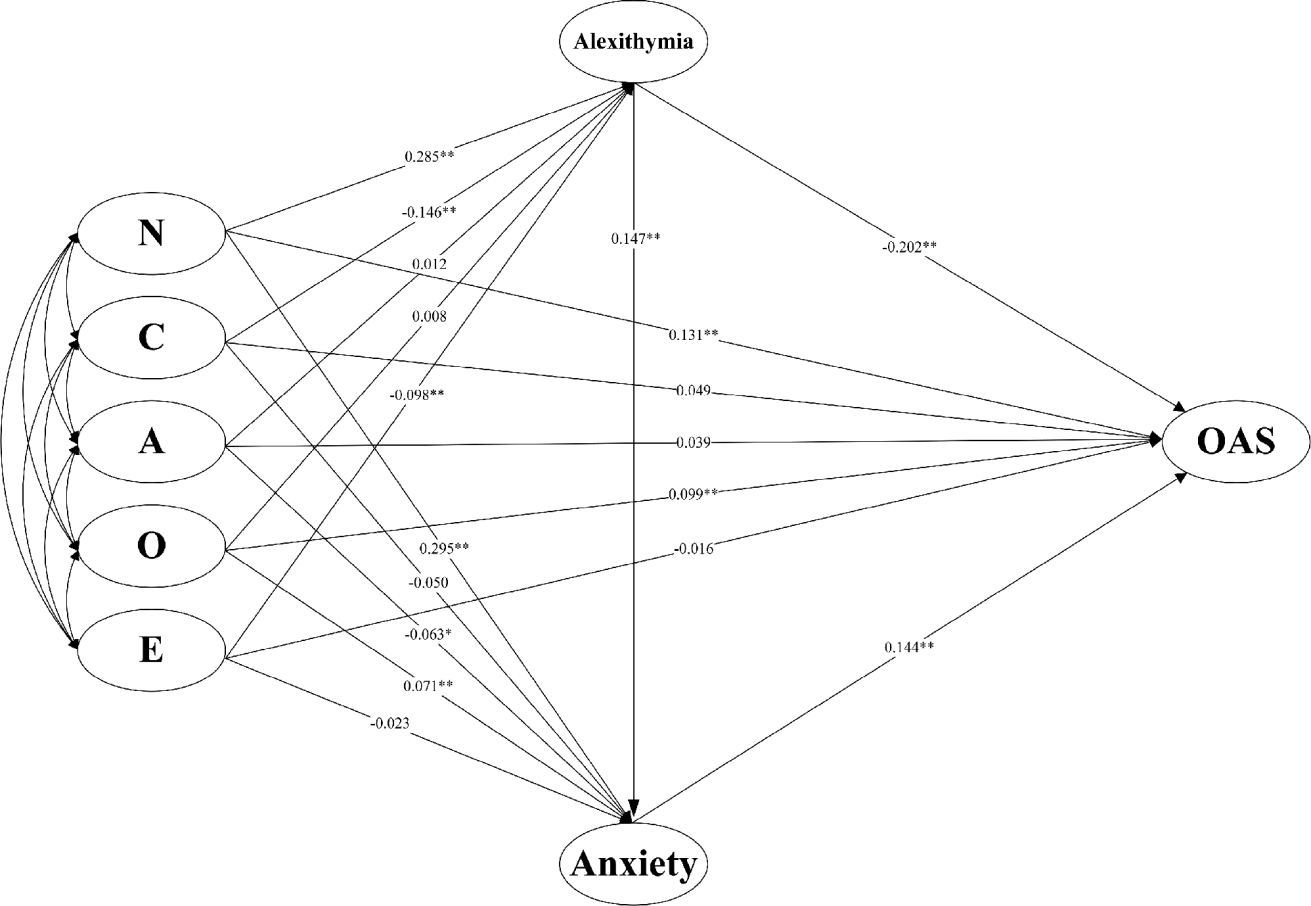
Mediation model and non-standardized path coefficients. *Note* OAS = Odor Awareness Scale; N = neuroticism; C = conscientiousness; A = agreeableness; O = openness; E = extraversion. *p < 0.05, **p < 0.01

Path analysis revealed that alexithymia was significantly predicted by neuroticism (*β* = 0.285, *p* < 0.01), conscientiousness(*β* = -0.146, *p* < 0.01), extraversion(*β* =-0.098, *p* < 0.05), but not by agreeableness (*β* = 0.012, *p* > 0.05) and openness (*β* = 0.008, *p* > 0.05). Anxiety, on the other hand, was significantly predicted by neuroticism (*β* = 0.295, *p* < 0.01), agreeableness (*β* =-0.063, *p* < 0.05), openness (*β* = 0.071, *p* < 0.01), and alexithymia (*β* = 0.147, *p* < 0.01), while conscientiousness (*β* = -0.050, *p* > 0.05) and extraversion (*β* = -0.023, *p* > 0.05) did not exhibit significant predictive power in this context. OAS was significantly predicted by neuroticism (*β* = 0.131, *p* < 0.01), openness (*β* = 0.099, *p* < 0.01), as well as alexithymia (*β* = -0.202, *p* < 0.01), and anxiety (*β* = 0.144, *p* < 0.01), while conscientiousness (*β* = 0.049, *p* > 0.05), agreeableness (*β* = 0.039, *p* > 0.05), and extraversion (*β* = -0.016, *p* > 0.05) showed no significant relationships (see Fig. 1).

The mediation effect test revealed that in the path from neuroticism to OAS, 95% confidence intervals (CI) of the total effect, direct effect, and all three mediation effects did not include 0, which indicates statistical significance, including the serial mediation effect (Effect = 0.006, Effect size = 4.6%, 95% CI [0.001, 0.014]). For the path from conscientiousness to OAS, the 95% CIs of both the total effect and the direct effect encompassed 0. However, two mediating effects had 95% CIs that did not include 0, indicating significant mediation. Specifically, alexithymia mediated the relationship between conscientiousness and OAS (Effect = 0.029, Effect size = 59.2%, 95% CI [0.011, 0.056]), and alexithymia and anxiety sequentially mediated the relationship between conscientiousness and OAS (Effect = -0.003, Effect size = 6.1%, 95% CI [-0.008, -0.001]). In the path from agreeableness to OAS, neither the total effect nor the direct effect was significant. However, one mediation effect was significant, with anxiety mediating the relationship between agreeableness and OAS (Effect = -0.009, Effect size = 23.1%, 95% CI [-0.023, -0.001]). In the path from openness to OAS, both the total effect and the direct effect were significant, with only one mediation effect being significant, where anxiety mediated the relationship between openness and OAS (Effect = 0.010, Effect size = 10.1%, 95% CI [0.004, 0.024]). In the path from extraversion to OAS, neither the total effect nor the mediating effects were significant. However, two mediation effects were significant, where alexithymia mediated the relationship between extraversion and OAS (Effect = 0.020, Effect size = 125.0%, 95% CI [0.007, 0.041]), and alexithymia and anxiety sequentially mediated the relationship between extraversion and OAS (Effect = -0.002, Effect size = 12.5%, 95% CI [-0.006, -0.001]) (more details in Table 2).

**Table 2:** Mediation paths and effect analysis

				Bootstrap 95%CI	
Path		Effect	Effect size	Lower	Upper
Total	N → OAS	0.122		0.077	0.159
Direct	N → OAS	0.131		0.070	0.196
Specific indirect	N → Alexithymia → OAS	-0.058	44.3%	-0.091	-0.029
	N → Anxiety → OAS	0.042	32.1%	0.018	0.073
	N → Alexithymia → Anxiety → OAS	0.006	4.6%	0.001	0.014
Total	C → OAS	0.068		-0.002	0.136
Direct	C → OAS	0.049		-0.020	0.124
Specific indirect	C → Alexithymia → OAS	0.029	59.2%	0.011	0.056
	C → Anxiety → OAS	-0.007	14.3%	-0.023	0.000
	C → Alexithymia → Anxiety → OAS	-0.003	6.1%	-0.008	-0.001
Total	A → OAS	0.028		-0.041	0.088
Direct	A → OAS	0.039		-0.029	0.098
Specific indirect	A → Alexithymia → OAS	-0.002	5.1%	-0.017	0.009
	A → Anxiety → OAS	-0.009	23.1%	-0.023	-0.001
	A → Alexithymia → Anxiety → OAS	0.000	0	-0.001	0.002
Total	O → OAS	0.108		0.053	0.167
Direct	O → OAS	0.099		0.043	0.156
Specific indirect	O → Alexithymia → OAS	-0.002	2.0%	-0.014	0.011
	O → Anxiety → OAS	0.010	10.1%	0.004	0.024
	O → Alexithymia → Anxiety → OAS	0.000	0	-0.001	0.002
Total	E → OAS	-0.002		-0.046	0.047
Direct	E → OAS	-0.016		-0.065	0.033
Specific indirect	E → Alexithymia → OAS	0.020	125.0%	0.007	0.041
	E → Anxiety → OAS	-0.003	18.8%	-0.012	0.001
	E → Alexithymia → Anxiety → OAS	-0.002	12.5%	-0.006	-0.001

*Note* OAS = Odor Awareness Scale; N = neuroticism; C = conscientiousness; A = agreeableness; O = openness; E = extraversion

## Discussion

To our knowledge, this is the first exploration into the relationship between the Big Five personality traits, alexithymia, anxiety symptoms, and odor awareness. Our analytical findings revealed a significant intercorrelation among them. Furthermore, it becomes evident that alexithymia and anxiety symptoms act as mediators between the Big Five personality traits and odor awareness. These observations lend robust support to the initial hypothesis outlined in the introduction section, namely predicated on the structure of the limbic system, an intrinsic association exists between personality, emotions, and olfaction, thereby leading to the association between personality traits, affective symptoms, and odor awareness.

The correlation analysis revealed that alexithymia was associated significantly with odor awareness, anxiety symptoms, and the various dimensions of the Big Five personality, aligning with prior research findings [14, 15, 58]. Existing studies have demonstrated that alexithymia bears not only relevance to psychiatric pathologies such as depression, anxiety, and somatization [76–81] but also to physical ailments like chronic pain, somatic illnesses, and obesity [82–84], which underscores alexithymia’s role as a wide-ranging pathological susceptibility factor. The significant correlation between alexithymia and the various dimensions of the Big Five personality may stem from alexithymia serving as a personality trait that intersects with certain aspects of the Big Five personality traits; some studies even propose that alexithymia represents a hybrid within the framework of the Big Five personality traits [85]. The negative correlation between alexithymia and odor awareness indicates deficits in emotional processing among alexithymic individuals [86–89], even through olfactory modalities [90]. Conversely, anxiety symptoms exhibited a positive correlation with odor awareness, implying that heightened anxiety levels are associated with increased olfactory attention, consistent with other research findings [15, 18, 19]. One explanation posits that anxiety sensitizes the sensory cortical system, enhancing sensory stimulus detection in the environment [91]. This pattern aligns with the clinical features of anxiety, where individuals maintain a heightened state of vigilance, hyperarousal, and preparedness to respond to sudden threats [92–94]. Anxiety symptom was positively correlated with neuroticism and negatively correlated with the other four personality dimensions, consistent with previous research [95, 96], although the correlation between anxiety and openness lacks statistical significance in this study. As Watson and colleagues [97] suggested, neuroticism represents a predisposition to experience heightened negative emotions, such as anger, anxiety, and depression.

This study marks the pioneering investigation into the relationship between odor awareness and the Big Five personality traits, uncovering significant positive correlations between odor awareness and neuroticism, conscientiousness, agreeableness, and openness. Prior studies have indicated that individuals with higher levels of neuroticism demonstrate greater olfactory recognition ability [52], while those with a prominent agreeableness trait exhibit heightened olfactory sensitivity [54], which may contribute to our findings partially. Odor awareness exhibited a negative correlation with extraversion, although this correlation is not statistically significant. Given that the various dimensions of the Big Five personality are not entirely independent [56, 98, 99] and display some degree of interrelatedness [59], it is also possible that other personality dimensions contribute to the relationship between odor awareness and extraversion. This study has also partially detected significant intercorrelations among the Big Five personality dimensions.

### Neuroticism

A structural model was scrutinized to assess the congruence of the data with the hypothesized framework. Given the interrelationships among the Big Five personality dimensions and other variables, we incorporated all dimensions of the Big Five personality into the model for analysis. This not only controlled for covariates but also rendered the research model more unified and parsimonious, thereby facilitating in-depth analysis.

Results supported the fit of the data according to the study’s model. Path analysis unveiled that heightened neuroticism predicted elevated alexithymia, implying that this personality dimension predisposes individuals to alexithymia to a certain extent, in line with prior research [58, 100]. In turn, individuals exhibiting heightened alexithymia scores grapple with difficulties in recognizing and describing their own emotions, potentially leading to diminished odor awareness. This highlights alexithymia’s role as a mediating factor in the relationship between neuroticism and odor awareness, a point further substantiated by the mediation effect test. Although the conventional definition of alexithymia underscores emotional disturbances, recent research has illuminated its influence extending to physiological responses, such as heart rate, arousal levels, and respiration [101, 102]. Therefore, alexithymia may impact an individual’s olfactory function, resulting in reduced attention to odors and consequently diminished odor awareness. This study also detected anxiety as a mediating factor between neuroticism and odor awareness. Existing researches have confirmed that individuals with high neuroticism tendencies are more prone to anxiety [56, 57]. Anxiety serves to sensitize the sensory cortical system [91], including the enhancement of olfaction. Clinical observations have revealed heightened olfactory sensitivity, reactivity, and odor awareness among anxious patients [18, 19] characterized by heightened vigilance and hyperarousal [92, 94, 103]. Similar results have been observed in non-clinical populations [15, 60].

Moreover, our investigation captured a sequential mediation effect of alexithymia and anxiety in the relationship between neuroticism and odor awareness. Research has indicated that individuals with high alexithymia tendencies are inclined to experience elevated anxiety symptoms [58, 104]. Specifically, individuals with high neuroticism exhibit emotional instability and inadequate emotional recognition and processing capabilities, likely leading to alexithymia. Those with high alexithymia are unable to resolve their emotion-related issues and difficulties, making them susceptible to anxiety. In turn, anxiety heightens sensory perception, including olfaction, ultimately resulting in increased odor awareness. Furthermore, this study established the significance of the direct effect, signifying that neuroticism can directly predict odor awareness. Individuals with high neuroticism tendencies tend to possess heightened odor awareness. Research has reported that individuals with high neuroticism scores can correctly identify a greater number of odors [52]. Olfactory neuroimaging studies have indicated that olfactory information processing largely relies on the neural anatomical structures within the limbic system, such as the amygdala, insula, and orbitofrontal cortex [105, 106]. Similarly, it has been posited that individuals with intense emotional reactions exhibit higher activation within the limbic system, which is associated with limbic structures [107, 108]. Given the pronounced interdependence between limbic structures and olfactory function, it is unsurprising that individuals with heightened emotional reactivity also pay considerable attention to odors.

Interestingly, the indirect effect of alexithymia on odor awareness was negative, while the direct effect of neuroticism on odor awareness was positive. Moreover, when incorporating anxiety symptoms, the indirect effect on this sequential path turned positive, demonstrating a complex phenomenon. This complexity arises because odor awareness, as an outcome variable, is influenced by various factors, with individual odor awareness being the result of the effects of all relevant factors. To be specific, in this study, individuals with high neuroticism tended to have high levels of alexithymia, which in turn directly predicted low odor awareness. Simultaneously, high levels of alexithymia contributed to high anxiety symptoms, and high anxiety symptoms positively predicted high odor awareness, akin to alexithymia indirectly predicting high odor awareness, which contrasted with the direct effect of alexithymia on odor awareness, creating a mutual antagonism, but not a mutual cancellation; in fact, these two effects varied in magnitude. The results indicated that the indirect effect size of alexithymia on the relationship between neuroticism and odor awareness (Effect Size = 44.3%) was much larger than the sequential indirect effect size of alexithymia and anxiety symptoms (Effect Size = 4.6%). However, the largest effect was the direct effect of neuroticism on odor awareness, ultimately resulting in a positive total effect. Overall, alexithymia can be considered as a suppressor of the effect of neuroticism on odor awareness, diminishing the total effect of neuroticism on odor awareness [109–111]. Despite Hayes asserts [112] that suppression or inconsistent mediation are merely labels for indirect effect phenomena rather than explanations, we employ such terminology only to elucidate the characteristics of alexithymia’s effects more clearly.

### Conscientiousness

Within the model, the total and direct effects of conscientiousness on odor awareness pathways did not attain statistical significance. However, we have discerned two significant mediating effects: alexithymia mediated the relationship between conscientiousness and odor awareness, and a sequential indirect effect involving alexithymia and anxiety as intermediaries between conscientiousness and odor awareness. Despite the nonsignificance of the total effect, recent scholarship has progressively coalesced around the consensus that a significant total effect is not an obligatory precondition for conducting mediation analysis [112–115]. High conscientiousness predicted low alexithymia, aligning with prior research findings [58, 100]. The conscientiousness dimension refers to self-regulation in both proactive and inhibitory aspects [116, 117]. Individuals high in conscientiousness exhibit proficiency in emotional recognition and regulation, rendering them less susceptible to alexithymia. Conversely, low alexithymia negatively predicted odor awareness and positively predicted anxiety, while anxiety positively predicted odor awareness. These contrasting effects on odor awareness, both positive and negative, may account for the nonsignificance of the total effect between conscientiousness and odor awareness. Similarly, as the indirect effect size of alexithymia (Effect Size = 59.2%) surpassed the sequential indirect effect size of alexithymia and anxiety symptoms (Effect Size = 6.1%), and the sign of the indirect effect of alexithymia aligned with the direct effect of conscientiousness on odor awareness, hence unlike neuroticism, alexithymia does not act as a suppressor between conscientiousness and odor awareness but rather functions as a conventional mediator [110, 111].

### Agreeableness

Regarding agreeableness, we detected only one significant mediating effect, namely, anxiety mediating the relationship between agreeableness and odor awareness. Agreeableness negatively predicted anxiety, in alignment with prior research findings [58]. Agreeableness represents a positive personality trait [56], characterized by high trust in others, a heightened sense of security, and a reduced susceptibility to anxiety, consequently resulting in lower attention to odors. However, within our analysis results, both the total and direct effects were positive values, albeit nonsignificant. In pairwise correlation analyses, agreeableness and odor awareness exhibited a significant positive correlation, albeit with a low correlation coefficient, suggesting that there may be a potential association between agreeableness and olfaction. Research has also uncovered a small yet significant positive correlation between agreeableness and olfactory sensitivity [54]. That study posits that the observed relationship between heightened olfactory sensitivity and agreeableness suggests individuals with high olfactory sensitivity tend to represent an increased interest in societal issues, including social odors (body odors). However, OAS primarily concerns odors in the environment, which may account for the nonsignificance of the total and direct effects in our results. Nevertheless, it is essential to acknowledge the potential presence of confounding factors or other mediating variables that could influence the relationship between agreeableness and odor awareness, necessitating further investigation in future research endeavors.

### Openness

In our study findings, we detected that openness exhibited a significant total and direct effect on odor awareness. Individuals with high openness demonstrate expansive interests and a proclivity for exploring novel experiences, places, and cuisines [116, 117]. Consequently, they may exhibit heightened attentiveness to odors. Furthermore, we also identified anxiety as a mediator in the relationship between openness and odor awareness. There exists subtle evidence suggesting a positive correlation between the fantasy facet of openness and anxiety [118]. An inclination toward fantasy thinking may lead to more frequent apprehensions and wishful thoughts rather than effective planning, thereby exacerbating anxiety and, consequently, affecting olfactory-related cognition.

### Extraversion

The study revealed that alexithymia serves as a mediating factor in the relationship between extraversion and odor awareness. Furthermore, we also found that alexithymia and anxiety acted as sequential mediators in the relationship. It aligns with prior studies [58, 119, 120] that extraversion negatively predicted alexithymia. Individuals with high levels of extraversion tend to experience a wide array of positive emotions and possess vivid emotional recognition and discrimination experiences [121, 122], which may lead to a reduced propensity for alexithymia. This may, in turn, provide a protective effect on odor awareness. However, due to the association between alexithymia and anxiety, a low level of alexithymia predicted a lower anxiety level, subsequently reducing sensitivity and attention toward odors. This may ultimately result in a nonsignificant total effect on the relationship between extraversion and odor awareness. The nonsignificant bivariate correlations between these variables also support this interpretation. Likewise, in the relationship between extraversion and odor awareness, the indirect effect size of alexithymia (Effect Size = 125.0%) much exceeded the sequential indirect effect size of alexithymia and anxiety symptoms (Effect Size = 12.5%). Furthermore, both the indirect effect of alexithymia and the direct effect were opposite in sign, thus, it can be posited that alexithymia acts as a suppressor in the relationship between extraversion and odor awareness [110, 111].

The current structural equation model stands as an appropriate and meaningful framework. While the present model elucidates a substantial portion of the relationships among the Big Five personality traits, alexithymia, anxiety symptoms, and odor awareness, certain associations remain inadequately explicated. Grounded in the limbic system, our theoretical framework provides a foundation for future research to delve more profoundly into the neurophysiological structures underpinning the relationships among personality, affective disorders, and olfaction. It is also warranted to incorporate additional variables for the investigation of their interrelations, such as other potential mediators like depression and social anxiety, and even exploring moderation effects [15].

The present study also bears certain limitations. Cross-sectional investigation cannot infer causality. The utilization of a convenience sampling method may introduce some bias, although it is a widely employed sampling approach. The relatively higher proportion of female participants in the study might contribute to the bias as well. There were more females in the classes surveyed. And with more than 70 items on the questionnaire and few rewards, males may be less willing to fill in the questionnaire than females, which may lead to the imbalance of gender ratio. However, the number of males in the sample was not low, and the t-test showed that gender had little influence on the dependent variables in the mediation model, so the bias caused by gender ratio was very small. Of course, future studies could improve the gender ratio. Our participants were all typical university students, which constrains the generalizability of the findings to a broader population. Future research could research more deeply into clinical populations to expand the scope of investigation.

## Conclusion

Collectively, the present study represents a first attempt to better understand the complex relationship between the big five personality, alexithymia, anxiety symptoms, and odor awareness. Specifically, it is also the first exploration into the association between the Big Five personality traits and odor awareness. Our investigation has illuminated that neuroticism stands as a conspicuous psychopathological risk factor, exhibiting a pronounced proclivity toward olfaction, with potential ramifications for impairments in emotional processing and a predisposition toward anxiety symptoms, thereby exerting an influence on individual odor awareness. Openness also bestows a certain degree of attention upon odors, and it inclines toward anxiety, consequently impacting individual odor awareness. Conscientiousness, agreeableness, and extraversion do not manifest discernible proclivities toward olfactory attention but exert indirect influences on odor awareness through their effects on affective symptoms. Particularly, alexithymia acts as a suppressor in both the relationship between neuroticism and odor awareness as well as in the relationship between extraversion and odor awareness, while playing as a conventional mediator in the relationship between conscientiousness and odor awareness. Rooted in the structural framework of the limbic system, where personality, emotion, and olfaction anatomically converge, our research findings endorse a certain degree of linkage between the Big Five personality traits, affective symptoms, and olfactory-related cognition. This study bolsters understanding of personality, emotion, and olfaction and lays a foundation for potential interventions and preventive measures in the clinical realm, addressing affective disorders and even olfactory dysfunctions.

## Data Availability

The datasets used during the current study are available from the corresponding author on reasonable request.
